# Whole Genome DNA Methylation and Gene Expression Profiling of Oropharyngeal Cancer Patients in North-Eastern India: Identification of Epigenetically Altered Gene Expression Reveals Potential Biomarkers

**DOI:** 10.3389/fgene.2020.00986

**Published:** 2020-10-08

**Authors:** Lastborn Marthong, Sahana Ghosh, Arindam Palodhi, Mohamed Imran, Neizekhotuo Brian Shunyu, Arindam Maitra, Srimoyee Ghosh

**Affiliations:** ^1^Department of Zoology, North Eastern Hill University (NEHU), Shillong, India; ^2^National Institute of Biomedical Genomics (NIBMG), Kalyani, India; ^3^Department of Otorhinolaryngology (ENT), North Eastern Indira Gandhi Regional Institute of Health and Medical Sciences (NEIGRIHMS), Shillong, India

**Keywords:** oropharyngeal cancer, DNA methylation, RNA-Seq, epigenetic regulation, transcription factor enrichment, biomarkers

## Abstract

Oropharyngeal cancer is a subtype of head and neck squamous cell carcinoma that is associated with unique risk exposures like consumption of smokeless tobacco and areca nut and is highly prevalent in the northeastern region of India, especially Meghalaya. However, the underlying epigenetic and transcriptomic changes in this cancer type is yet to be delineated. We have undertaken a study on genome wide somatic alterations in the DNA methylation and transcriptome in oropharyngeal cancer patients from this region using genome wide techniques in paired tumors and adjacent normal tissues. By using integrative approaches, we have identified 194 epigenetically silenced and 241 epigenetically overexpressed genes in the tumor tissue of these patients. Pathways that are significantly enriched by these genes include the pathways of immune systems, such as the interleukin signaling pathways and Toll-like receptor signaling pathway. Also, osteoclast differentiation pathway was found to be epigenetically upregulated. The pathways enriched by the epigenetically downregulated genes were found to be predominantly those involved in xenobiotic metabolism and keratinization. Two major transcription factors – *SPI1* and *RUNX1* were identified as epigenetically dysregulated, which further modulates 129 downstream genes. Comparison of our observations with the head and neck cancer data from TCGA revealed distinct DNA methylation and gene expression landscapes which might be specific for oropharyngeal cancer. HPV DNA sequences were not detected in any of the tumor samples in RNA-Seq data. The results obtained in this study might provide improved understanding of the disease.

## Introduction

Oropharyngeal cancer is a type of head and neck cancer (HNC) that develops in the oropharynx, which includes the soft palate, the tonsils, lingual tonsils, the base of the tongue and the posterior pharyngeal wall. Morphologically, squamous cell carcinomas (SCCs) are the most common type of oropharyngeal cancer. In 2018 alone, there were 92,887 new cases of oropharyngeal cancer and 51,005 attributed deaths worldwide (Bray et al., 2018). India has the highest incidence as well as mortality rate for oropharyngeal cancer, with 17,903 new cases and 14,953 deaths reported for 2018 (Bray et al., 2018).

The etiological factors associated with the multifactorial development of oropharyngeal cancer include consumption of tobacco (smoking and smokeless), areca nut and alcohol (Herrero et al., 2003). Cases due to Human Papilloma Virus (HPV) infections have also been on the rise in recent years (Chaturvedi et al., 2011). The higher incidence rates of oropharyngeal cancer in India, especially in northeast India (Dikshit et al., 2012), has been correlated to their specific dietary and lifestyle habits such as chewing of betel nut, consumption and exposure to smokeless tobacco (Taranikanti and Das, 2015; Adhikari and De, 2016). It has also been found to be more prevalent among poor socio-economic groups, lower educational and lower income groups (Sharma et al., 2018).

DNA Methylation is a stable molecular alteration of DNA, which occurs early and commonly in cancer, is easy to detect in small amounts and is known to have potential in predicting survival differences and/or responses to therapy (Jones and Laird, 1999; Esteller, 2008; Jones and Baylin, 2002). The identification and characterization of differentially methylated genes and regions could be immensely useful in the early detection of cancers leading to more effective diagnosis and better treatment outcomes (Kulkarni and Saranath, 2004; Towle and Garnis, 2012; DegliEsposti et al., 2017; Leygo et al., 2017). Recent candidate gene studies have implicated a number of genes involved in tumor development. *P16*, *MGMT*, *DAPK*, *RASSF1*, *MINT31*, *EDNRB*, *KIF1A*, *DCC*, *P15*, *hMLH*1 and *E-cadherin* genes in conjunction with HPV infection and tobacco usage have been correlated to oral cancer and other head and neck cancers among the Indian population (Merlo et al., 1995; Viswanathan et al., 2003; Kaur et al., 2010; Choudhury and Ghosh, 2015). A recent study on the whole genome methylation profile for oral cancer was undertaken on a subset of the northeast India population (Khongsti et al., 2017). However, no whole genome, candidate gene methylation or gene expression studies have been conducted on oropharyngeal cancer in India.

## Materials and Methods

### Sample Collection

The samples were collected from biopsy procedures of the department of ENT, North Eastern Indira Gandhi Regional Institute of Health and Medical Sciences (NEIGRIHMS), Shillong, Meghalaya during the period of August 2015 to June 2017, conducted by trained professionals. All samples were from the northeast population of India including the respective indigenous tribes of the region. Tumor and adjacent normal tissue samples were collected from 16 treatment-naive oropharyngeal cancer patients and only tumor samples were collected from 10 treatment-naive oropharyngeal cancer patients. The samples were directly transferred to vials containing RNA*later*^TM^ Stabilization Solution which were then stored in −80°C until further requirement. Written informed consent was obtained from all participants. The research protocol was approved by the Institutional Ethics Committees of North Eastern Hill University, Shillong, Meghalaya (IECHSP/2015-16 dated 23 June 2017), North Eastern Indira Gandhi Regional Institute of Health and Medical Sciences, Shillong, Meghalaya (NEIGR/IEC/2015/0039 dated 21 April 2016) and National Institute of Biomedical Genomics, Kalyani (Dated 12th June 2015).

### Extraction of DNA and Total RNA

Genomic DNA and total RNA were isolated from oropharyngeal tumor and adjacent normal tissue using AllPrep DNA/RNA Mini Kit (Qiagen).

### Bisulfite Conversion of Extracted DNA and DNA Methylation Profiling by Infinium 450K Methylation Assay

For DNA methylation assay, 850 ng of each DNA sample was bisulfite converted using EZ DNA methylation kit (Zymo Research) following the manufacturer’s protocol. After Bisulfite conversion, whole genome DNA methylation assay of tumor and paired adjacent normal samples from 16 oropharyngeal cancer patients and tumor samples from another 10 independent oropharyngeal cancer patients was performed using an Illumina Infinium HumanMethylation450 BeadChip (Bibikova et al., 2011), that interrogates 485,577 CpG sites per sample, following manufacturer’s protocol. The processed chips were scanned using an iScan reader (Illumina) and raw data were obtained as intensity Data Files (^∗^.idat) format. The raw data were quality checked using the methylation module (v1.8.5) of GenomeStudio software v2010.3 (Illumina).

### Analysis of Epigenome Wide DNA Methylation Data of Orapharyngeal Carcinoma

Raw IDAT files were exported to R package “minfi” (Aryee et al., 2014) to perform data analyses using the discovery pipeline. The methylation intensity for each of the ∼485,000 CpG sites was calculated as β (beta) value which represents the quantitative measure of DNA methylation. β value is calculated as the ratio of fluorescent signals from the methylated probes to the sum of the signals from the methylated and unmethylated probes as β = [M/(M + U + 100)], where M = methylated probe and U = unmethylated probe. The β value ranged from 0 to 1, while unmethylated probes had a β-value close to 0 and fully methylated probes had a β-value close to 1 (Bibikova et al., 2011; Khongsti et al., 2017; The Cancer Genome Atlas Network, 2015).

Samples with 25% or more CpG sites having a detection of *P*-value(s) >= 0.01 were dropped. Further to minimize the chance of getting false positive results, we filtered out CpG probes with: detection *P*-value(s) >= 0.01 present in 50% or more samples; any probe with “NA”- masked values; polymorphic (MAF > 0.01) SNPs (dbSNP build 150) located within 10 bp of the interrogated CpG site; any non-CpG sites; CpG site located on sex chromosome (Das et al., 2019). Misclassified samples were identified using a multidimensional scaling plot over beta values of 1,000 of the most variable CpG sites and hierarchical clustering. We identified 2 pairs of misclassified samples and removed them. Two types of normalization procedure were performed: (1) Subset quantile within array normalization (SWAN) (Maksimovic et al., 2012) to remove bias due to usage of two types of infinium chemistry and (2) Quantile normalization (over beta value) to remove inter sample randomness.

Identification of CpG sites was performed using the method described in detail in the Supplementary Material. The CpG sites with Benjamini-Hochberg corrected *p*-value <0.05 and average |Δβ| ≥0.2 (tumor vs adjacent normal) were considered as significantly differentially methylated CpG sites (DMPs). A CpG site was considered hypermethylated if average Δβ ≥ 0.2 or hypomethylated if average Δβ≤−0.2. DMPs were further mapped to genes to identity differentially methylated regions. Within gene boundaries the regions considered were promoter (TSS1500, TSS200, 5′UTR), gene body (intron & exon) and 3′UTR. Significantly differentially methylated region (DMRs) were defined as the genomic regions having at least 25% unidirectional DMPs (The Cancer Genome Atlas Network^∗^, 2015). The methylation level of DMRs were quantified as average β values of DMPs mapped to it.

### Verification of Identified DMRs by a Parallel Approach

Due to an insufficiency of suitable samples for downstream confirmation experiments, a Supplementary Data analysis tool was used to verify the results obtained over the primary analysis. The data was analyzed using an alternative method to identify differentially methylated loci and compare the overlap between the results obtained using these two methods. The raw intensity data files (^∗^.idat) were processed by Illumina GenomeStudio methylation module and average β values were generated which were then further imported to Illumina Methylation Analyzer (IMA) (Wang et al., 2012) to identify DMR. Wilcoxon signed-rank test was performed to detect genomic regions with significantly altered DNA methylation between tumor and adjacent normal samples. A region is considered as DMR having Benjamini-Hochberg adjusted *p*-value <0.05 (details of method provided in the Supplementary Material).

### Sequencing of Whole Transcriptome

Quality control and quantification of the extracted RNA was performed using NanoDrop^TM^ 2000 (Thermo Fischer Scientific) spectrophotometer and libraries were assessed by RNA Nano chip (Agilent) in 2100 Bioanalyzer (Agilent). RNA samples with OD 260/280 ratio ≥2 and RNA Integrity Number (RIN) ≥ 0.7 are selected for sequencing library preparation. The selected whole RNA samples were depleted for rRNA using the Ribo-Zero^TM^ Magnetic Kit (Illumina) according to the manufacturer supplied protocol. The purified RNA samples were used for preparation of sequencing libraries using TruSeq RNA Library Prep Kit v2 (Illumina) following the manufacturer supplied protocol. The sequencing libraries with unique barcodes prepared from each sample were pooled before loading in Illumina HiSeq – 2500 to generate 2 × 100 bp reads using Illumina TruSeq SBS V3 sequencing kit.

### Analysis of Whole Transcriptome Sequence Data of Oropharyngeal Cancer Patients

The QC passed sequence reads in FASTQ format were aligned to the human reference genome (UCSC hg19) and the corresponding annotation file from Ensembl using TopHat2 (Trapnell et al., 2012). The accepted mapping reads were processed through the *Cufflinks* (Trapnell et al., 2012) package to identify the differentially expressed genes. Briefly, *Cufflinks* was used to assemble the transcripts post alignment and *Cuffmerge* was used to merge several assemblies of transcripts produced by cufflinks. Then *Cuffnorm* was applied on the alignment file produced by TopHat2, using the merged transcript file produced by *Cuffmerge* as annotation and normalized expression values (in FPKM- Fragments Per Kilobase of transcript per Million mapped reads) of all the expressed genes across all the samples (both tumor and adjacent normal) were obtained. Identification of differentially expressed genes across tumor and adjacent normal tissue was performed using *Cuffdiff* module of Cufflinks.

### Pathway Enrichment Analysis

Epigenetically silenced and upregulated genes selected for pathway analysis were imported into the Cytoscape (Shannon et al., 2003). Both KEGG and REACTOME pathway enrichment analyses were performed with ClueGo V2.5.1 (Bindea et al., 2009) and Cluepedia V1.5.1 (Bindea et al., 2013) plugin of Cytoscape software. ClueGo visualized the pathway in a functionally categorized network and Cluepedia was used to visualize the associated genes within the network. For the analysis, a two-sided hyper-geometric test was used and followed by the Bonferroni step down correction to correct the *p*-value for the terms and groups created by ClueGO. The Kappa score threshold value was set to ≥0.4. Only the pathways resulted with *P*-value <0.05 were considered significant.

### Transcriptional Regulatory Network

ENCODE (The Encode Project Consortium, 2012) and the ChEA consensus transcription factor dataset (Lachmann et al., 2010) from the Enrichr (Kuleshov et al., 2016) library was taken to construct the Transcriptional Regulatory Network. The dataset contains 93 unique transcription factors and their putative target genes as a list. Epigenetically silenced and overexpressed genes were compared with the target gene lists in the data and their respective transcription factors were taken. The compared list contains transcription factors targeting the selected genes used in Cytoscape for network visualization and analysis. Centrality measures were calculated by NetworkAnalyser tool (Assenov et al., 2007) in the Cytoscape. Transcription factors having high out-degree value i.e., transcription factors targeting higher number of differentially expressed genes, were considered as significant transcription factors.

## Results

### DNA Methylation Alterations in Oropharyngeal Tumors

DNA methylation analysis revealed specific aberrant alterations in oropharyngeal squamous cell tumors. A total of 14 pairs of tumor and adjacent normal tissues (discovery set) and 10 additional tumor tissues from independent patients (validation set) were used for global methylome analysis using Illumina Infinium Human Methylation 450k assay. Analysis of the discovery set of samples identified 25,494 significantly differentially methylated CpG loci (DMP), each DMP displaying average |Δβ| ≥ 0.2 and adjusted *P*-value <0.05 (Supplementary Table 1). We found a strong correlation between the β values of these 25,494 DMPs in the tumors of the discovery set and the same in tumors of the validation set (Spearman rho = 0.86 and *P*-value < 2.2e-16) (Supplementary Figure 1). Out of these DMPs, 63% (*n* = 16,015) were found to be hypermethylated in the tumor compared to adjacent normal tissue while 37% (*n* = 9,479) were found to be hypomethylated. The methylation patterns observed in the CpG islands (CGI) including associated region (shore & shelf) and those observed in non-CGI open sea region revealed distinct differences. We found that ∼84.5% (*n* = 2441) of the DMPs in the CGI along with shore & shelf were hypermethylated whereas ∼48% (*n* = 5403) in the non-CGI open sea were hypermethylated. Similarly, ∼15% (*n* = 445) of the DMPs in the CGI were hypomethylated while ∼52% (*n* = 5856) of the non-CGI open sea region were hypomethylated.

**FIGURE 1:**
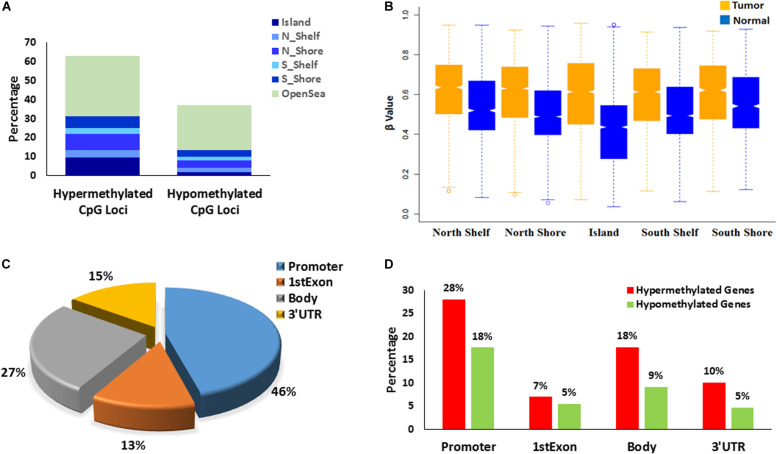
Characterizations of differentially methylated CpG probes/positions: DMPs (*N* = 25,494) and differentially methylated regions/genes: DMRs (*N* = 5,058). **(A)** Distribution of the hyper-methylated and hypo-methylated DMPs across different CGI regions and open sea. **(B)** DMPs mapped to different CGI regions (*N* = 11,422) depicts Islands gains maximal methylation in tumor tissues that gradually reduces to Shore. **(C)** Epigenomic localization of DMRs in different genomic regions – promoter, 1st exon, gene body and 3′ UTR. **(D)** Distribution of hyper-methylated and hypo-methylated DMRs across different genomic regions.

We found that 18,219 DMPs mapped to 7,133 genes (which includes promoter, 5′UTR, gene body consisting both intron and exon and 3′UTR). Of these, ∼94% (*n* = 6696) were found to be protein coding genes. A gene region that had at least 25% of the unidirectional DMPs mapped to it, was designated as a differentially methylated region or gene (DMR). About 56% (*n* = 4,004) of these 7,133 genes were identified as DMRs (Figure 1).

### Differential Promoter Methylation

Since differential DNA methylation in the promoter regions of genes impact on gene expression, we next investigated the promoter DNA methylation. A gene promoter was defined as a region which consisted of 1,500 bp on either side of the transcription start site (TSS) that included the TSS1500 (1500 bp upstream of TSS), TSS200 (200 bp upstream of TSS) and 5′UTR regions (Lokk et al., 2014). TSS200 is important for the binding of transcription factors which ultimately affects transcription. Approximately 35% (*n* = 6,409 of 18,219) of DMPs were mapped to the promoter region of genes and ∼58% (*n* = 2316) of protein-coding genes carrying DMRs were found to be differentially methylated in gene promoters based on the DMR analysis. Out of these, promoters of ∼61% (*n* = 1,421) genes were found to be hypermethylated while those of ∼39% (*n* = 895) genes were found to be hypomethylated.

**FIGURE 2:**
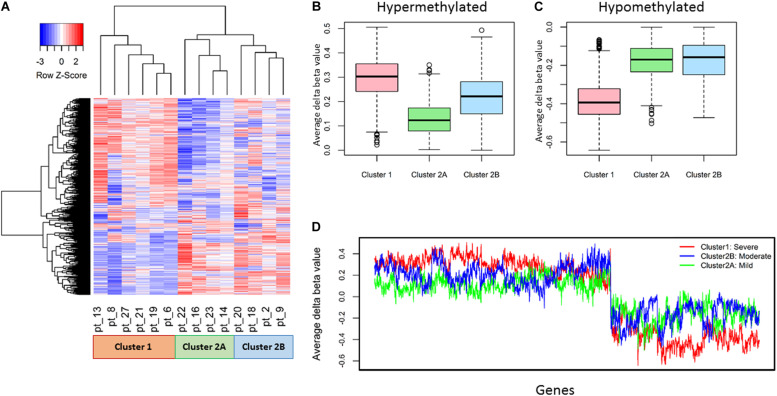
Representation of differential DNA methylation profile (Δβ) of 2,316 promoter genes in 14 patients. **(A)** Unsupervised hierarchical clustering and heatmap classified 14 OPSCC patients into two major clusters, further cluster 2 consisted of two sub clusters. **(B,C)** The ranges of average Δβ values clearly elucidates that DNA methylation alternations found in Cluster 1: severe, Cluster 2A: mild, Cluster 2B: moderate respectively from box and whisker plot. **(D)** The cluster wise distribution of average Δβ values of each gene depicts extreme level of hyper and hypo methylation in cluster 1 whereas cluster 2A shows minimal DNA methylation alterations.

The 14 patient samples were classified into two clusters based on the DNA methylation profile (Δβ) of the 2,316 genes; #1, with severe DNA methylation changes, and #2. Further, Cluster #2 consisted of two sub clusters 2A and 2B with moderate and mild DNA methylation changes respectively (Figure 2).

Overall, we identified 74 genes which were significantly differentially methylated in all the promoter sub regions that includes TSS1500, TSS200 and 5′UTR (Supplementary Table 1). Hypermethylation was observed in 42 genes and 32 were hypomethylated.

### Verification of Identified DMRs by a Parallel Approach

**FIGURE 3:**
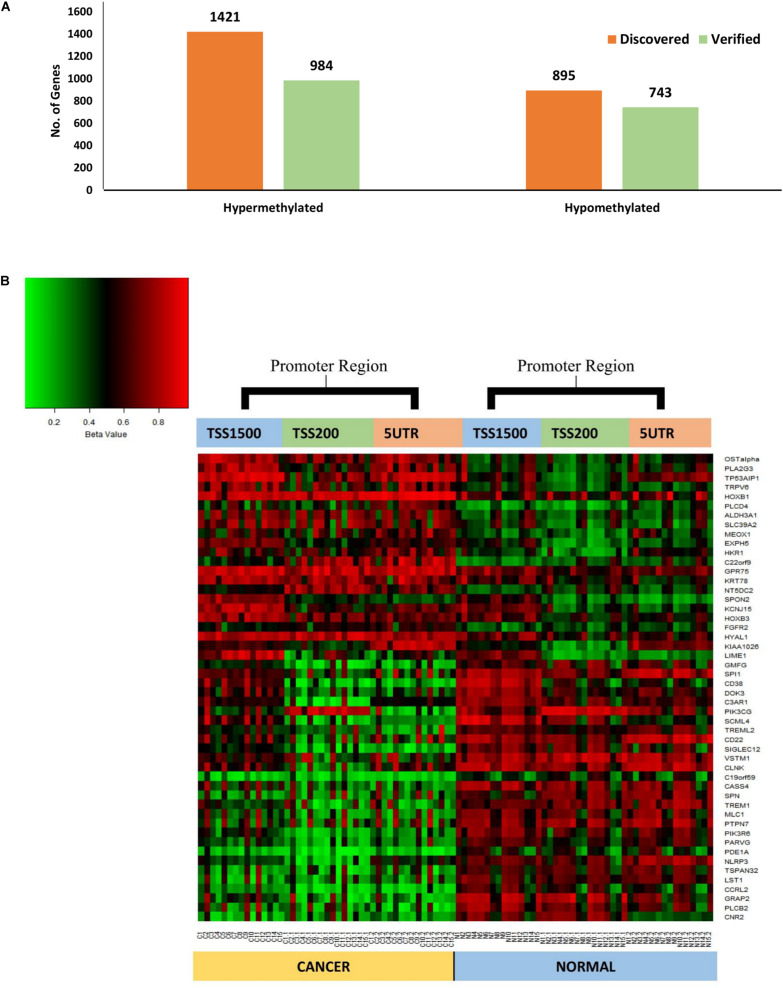
**(A)** Chart showing the comparison between the number of discovered and validated genes, 69% (*n* = 984) and 88% (*n* = 743) of hypermethylated and hypomethylated genes respectively, were verified. **(B)** Heat map of the common significantly differentially methylated genes (*n* = 50) in the promoter region (TSS1500, TSS200 and 5‘UTR) showing clear differential methylation between tumor and matched adjacent normal tissues. Unmethylated (0%) are represented by green whilst fully methylated (100%) are in red.

The parallel analysis of DNA methylation data identified ∼75% (*n* = 1727) of genes that were commonly differentially methylated in the promoter region. Approximately, ∼69% (*n* = 984) of hypermethylated genes were verified, whereas ∼83% (*n* = 743) of hypomethylated genes were verified. Overall, ∼52% (*n* = 22) of hypermethylated and ∼88% (*n* = 28) of hypomethylated genes were verified respectively (Figure 3) which were uniformly differentially methylated in TSS1500, TSS200 and 5′UTR regions that entirely constitutes the gene promoter (Supplementary Table 2).

### Comparison With TCGA Data

We compared the promoter DNA methylation alterations obtained by us in these OPSCC patients with that of the publicly available TCGA HNSCC data of 528 patients of HNSCC (The Cancer Genome Atlas Network^∗^, 2015). We found an overlap of 50 hypermethylated genes between our gene list and the 843 genes reported by TCGA on HNSCC patients. We also compared our data with that of OPSCC patients (78 patients) in the TCGA study. We found a strong correlation between the hypermethylated and hypomethylated genes detected in our dataset and the TCGA data on OPSCC patients (Spearman’s rank correlation ρ = 0.8; *p*-value < 2.2 × 10^–16^ for hypermethylated genes and ρ = 0.9; *p*-value < 2.2 × 10^–16^ for hypomethylated genes) (Supplementary Figure 2).

### Gene Expression Alterations Associated With Oropharyngeal Cancer

**FIGURE 4:**
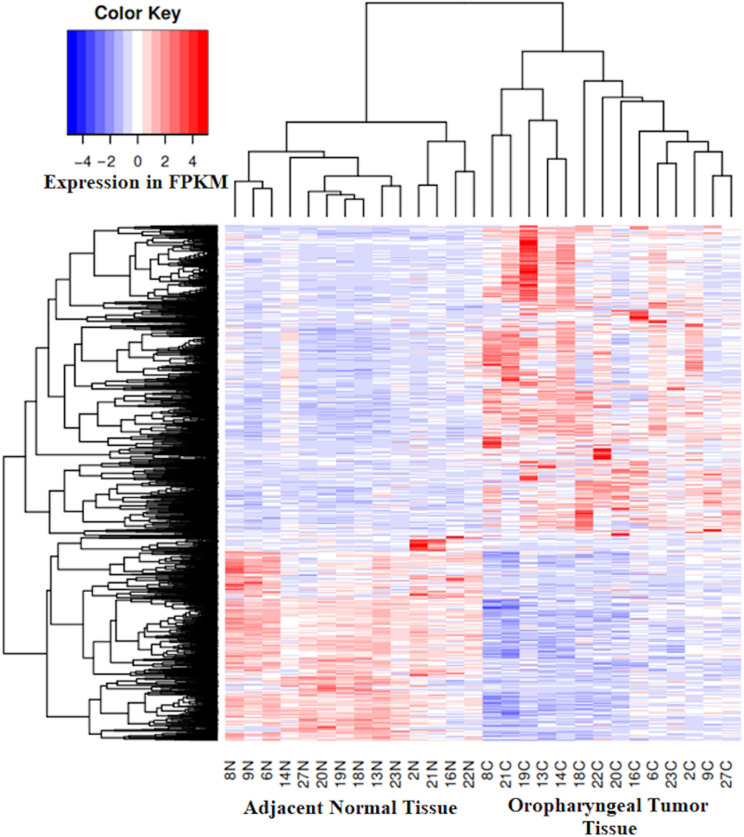
Expression heatmap of 3,120 genes in the tumor tissue and the matched adjacent normal tissue shows overexpressed (1,850 genes, Red) and underexpressed (1270 genes, Blue) genes in the tumor tissue from oropharyngeal cancer patients with respect to paired adjacent normal tissue.

Alterations in gene expression patterns in oropharyngeal squamous cell carcinoma was identified by RNA-Seq of paired tumors and adjacent normal tissue samples obtained from 14 patients, for whom whole genome DNA methylation assay was also performed. In the tumor tissue, an average of 27,248 genes were found to be expressed per patient (Supplementary Table 3), with pt27 expressing the highest (30,335 genes) and pt19 expressing the lowest number of genes (22,842 genes). On average, 26,855 genes were found to be expressed per patient in the adjacent normal tissue with pt16 expressing highest (29,437 genes) and pt8 expressing the lowest number of genes (22,676 genes). We have identified 3,120 genes differentially expressed in tumor tissue with respect to the adjacent normal tissue (Supplementary Table 4). Among these, the expression of 1,270 genes were downregulated and 1,850 genes were upregulated in the tumor when compared to adjacent normal tissue (Figure 4). The *FSHR* gene was found to be most underexpressed in tumor tissue along with keratinization genes such as *KRT76*, *KRT3* and *MUC7*. Several immune response regulatory genes, such as *IL6*, *IL8*, *CXCL5* etc. were found to be overexpressed in tumor tissue (Supplementary Table 4). None of the samples harbored sequence reads which mapped to HPV genomic sequences.

### Epigenetic Regulation of Gene Expression in Oropharyngeal Cancer

**FIGURE 5:**
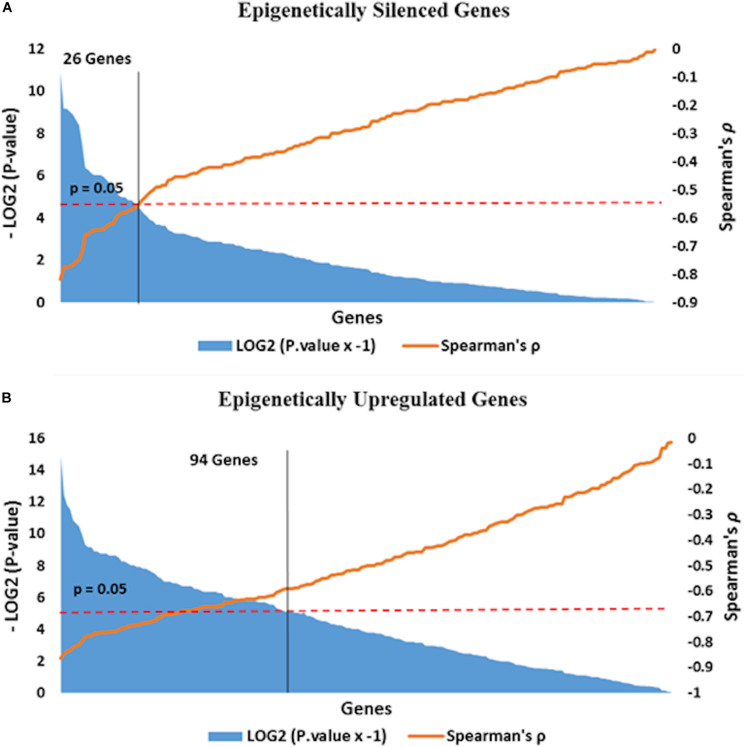
Epigenetically **(A)** silenced and **(B)** overexpressed genes are distributed along the *X*-axis according to Spearman’s correlation value in the ascending order. Negative correlation value represents the opposite direction of β value and log2 fold change expression values. Correlation test *P* value threshold (>0.05, red line in the plot) demarcates genes (yellow line in plot) with statistically correlated promoter methylation driven expression.

We performed integrative analysis of our data on DNA methylation in the promoter region and alteration in gene expression in tumor tissue with respect to adjacent normal tissue, to identify epigenetically regulated genes in oropharyngeal squamous cell carcinoma. Genes with reduced expression as well as promoter hypermethylation in tumor tissue compared to adjacent normal tissue were defined as epigenetically silenced genes. Genes exhibiting overexpression as well as reduced promoter methylations in tumor tissue compared to adjacent normal tissue were defined as epigenetically overexpressed genes. In this study we have found 194 epigenetically silenced and 241 epigenetically overexpressed genes in tumor tissue (Supplementary Table 5). Further, we calculated Spearman’s correlation to identify genes having significantly correlated promoter methylation and expression status. Epigenetic silencing of 27 genes (Spearman’s ρ < −0.5, *P* < 0.05) and epigenetic overexpression of 94 (Spearman’s ρ < −0.5, *P* < 0.05) genes were found to be corrected with statistical significance (Figure 5).

### Enrichment of Epigenetically Silenced and Overexpressed Pathways

**FIGURE 6:**
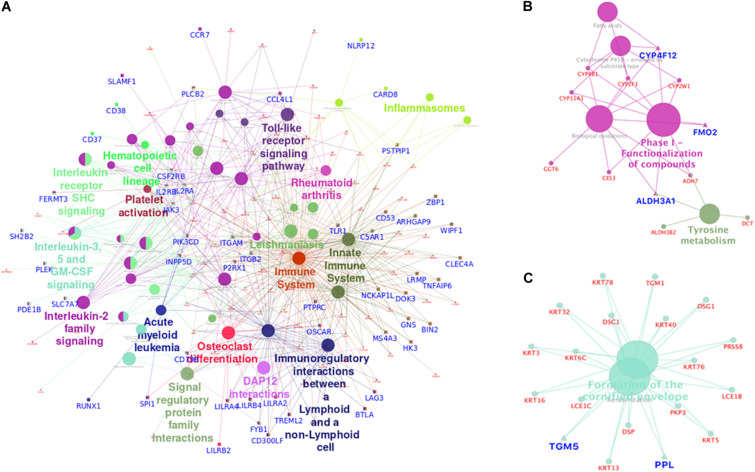
Functionally grouped network visualization for epigenetically upregulated and downregulated genes. Each Node represents a pathway and the node size represents the particular term’s enrichment significance (*P*-value). Genes highlighted in blue are of epigenetically significant genes. Epigenetically upregulated genes were found to be over-represented in immune system pathways and osteoclast differentiation pathways indicating immune evasion and bone marrow invasion **(A)** potential of tumor derived oropharyngeal cancer cells. Enrichment of epigenetically silenced genes in the xenobiotic metabolism **(B)** pathway suggests reduced carcinogen tolerance in oropharyngeal cancer cells. Also epigenetic down regulation of keratinization **(C)** pathway related genes indicate escape from growth inhibition by cell to cell adhesion.

Overall, 51 pathways were significantly enriched for the differentially expressed genes which were epigenetically controlled (*n* = 435). Using ClueGo (Bindea et al., 2009), these pathways were functionally grouped and visualized as networks. Epigenetically upregulated genes (*n* = 241) were found to be enriched in immune-regulation and osteoclast differentiation pathways. The pathways of immune systems, such as the interleukin signaling pathways and Toll-like receptor signaling pathway were significantly overrepresented in this group. The osteoclast differentiation pathway, represented by genes such as *SPI1*, *OSCAR* and *LILRB2*, was also significantly overrepresented (Figure 6A). The pathways enriched by the epigenetically downregulated genes (*n* = 194) were found to be predominantly involved in xenobiotic metabolism and keratinization (Figures 6A–C). Xenobiotic metabolizing genes such as *CYP4F12* and *ALDH3A1*; keratinization gene *PPL*; and *FGFR2* genes are some of the important genes represented in the pathways.

### Transcription Factor Prediction

**FIGURE 7:**
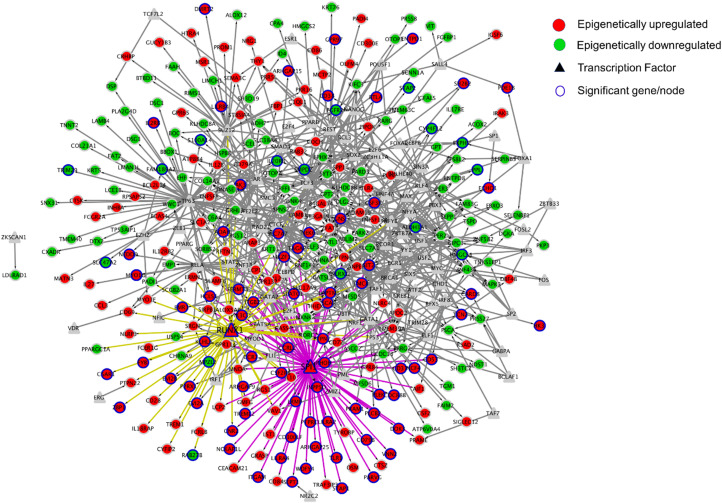
Network visualization of Transcriptional Regulatory Network created using Cytoscape. In this study, Nodes represent the TFs and genes, whereas the edges represent the regulation between TFs and genes. Out of 435 differentially expressed and epigenetically regulated genes (241 upregulated and 194 silenced) identified in this study, transcription factors SPI1 and RUNX1 were predicted to be associated with 81 and 48 genes respectively. Fifty-three genes were predicted to be regulated by transcription factor TP63, which itself was not differentially expressed in tumor tissue in oropharyngeal cancer.

We identified transcription factors which regulate the greatest number of differentially expressed genes. We used the ENCODE and ChEA TF consensus dataset from Enrichr; as the combination of these two datasets improved the TF prediction. This Transcriptional Regulatory Network contained 382 nodes (genes) and 839 edges (interactions). *SPI1* had the highest out-degree value (81 edges) followed by *TP63* (53 edges) and *RUNX1* (48 edges) (Figure 7). Among the top three transcription factors having higher out-degree value, *SPI1* and *RUNX1* were found to be epigenetically regulated (Figure 7).

## Discussion

Epigenetic modifications, particularly DNA methylation changes, play a critical role in cancer development. We undertook this genome wide DNA methylation and gene expression study on paired tumor and adjacent normal samples from patients of oropharyngeal cancer, a unique subtype of head and neck cancer, in a region in India, which has one of the highest prevalence for the same (Cancer Atlas of India). OPSCC from this region is associated with typical risk exposures i.e., consumption of areca nut with betel leaf and tobacco. Previous reports indicate that areca nut influences DNA methylation in squamous cell epithelium (Lai et al., 2014).

In our study, whole genome DNA methylation profiling analysis using the discovery pipeline identified 25,494 significantly differentially methylated CpG loci (DMP), of which 63% were hypermethylated and 37% were hypomethylated. This is in line with previous observation in other cancer types (Das et al., 2019) where hypermethylation of promoter CpGs and hypomethylation of gene body CpGs have been found. Approximately 35% (*n* = 6,409 of 18,219) of DMPs were mapped to promoter region of genes and ∼58% (*n* = 2316) of protein-coding genes were found to be differentially methylated in gene promoters based on the DMR analysis. The results of the DNA methylation analysis were verified using another analysis pipeline. RNA-Seq performed on these paired samples led to the identification of 3,120 differentially expressed genes in tumor tissue with respect to the adjacent normal tissue. Among these, expression of 1,270 genes was downregulated and that of 1,850 genes was upregulated.

Integration of the DNA methylation and gene expression results were undertaken by us to identify genes which were differentially expressed due to changes in DNA methylation alone. We found that 194 genes were epigenetically silenced, and 241 genes were epigenetically overexpressed in tumor tissue of OPSCC. Epigenetically upregulated genes (*n* = 241) were found to be enriched in immune-regulation and osteoclast differentiation pathways. The upregulation of genes belonging to the immune regulation pathway, in particular interleukin and Toll-like receptor signaling genes, might reflect anti tumor immune activation in these patients. TLRs have been reported to induce autophagy and programmed necrosis of tumor cells as well as to activate cytokines and anti tumor CTL response in patients (Cen et al., 2018). One of the epigenetically upregulated genes in these oropharyngeal tumors, the Leukocyte immunoglobulin-like receptor B2 (LILRB2), has been shown to be overexpressed and is a tumor promoting gene in HCC as well (Li et al., 2018). These findings might have important consequences on novel immune therapeutic possibilities in OPSCC. Recently reported studies have shown that bone resorption by osteoclasts is involved in the invasion of malignant cancer cells (Guise and Mundy, 1998; Jimi et al., 2011) and particularly in head and neck cancer (Sambandam et al., 2018). Our results indicate that osteoclast differentiation might be involved in tumor growth in OPSCC. The epigenetic upregulation of the proto-oncogene SPI1 and the Osteoclast Associated Ig-Like Receptor (Oscar) are also significant findings for this cancer type samples as upregulation of these genes would promote bone invasion of cancer cells. The pathways enriched by the epigenetically downregulated genes (*n* = 194) were found to be predominantly those involved in xenobiotic metabolism and keratinization. The downregulation of the xenobiotic metabolizing enzyme like *CYP4F12* is in agreement with the findings of Masood et al. (2011). Hypermethylation mediated downregulation of *ALDH3A1* is in correlation with the findings of Poage et al. (2012) who reported it to be a biomarker of HNSCC. Transcription factor prediction analysis of our integrated results led to the identification of *SPI1* and *RUNX1* as important transcription factors in this network. *SPI1* encodes PU.1, a transcription factor that has been found to be involved in progress in various cancer types (Xu et al., 2018). *RUNX1* has been reported to be a driver in cancer types (Wu et al., 2015) and a member of the Runt family, *RUNX3*, has been found to have an oncogenic role in head and neck cancer (Kudo et al., 2011). These observations might be useful in developing new therapeutic targets and new methods of predicting malignant behavior.

The overall overlap between the differentially methylated genes between TCGA-HNSCC (The Cancer Genome Atlas Network^∗^, 2015) and our studies is apparently low (only ∼6%). However, this can be explained by the fact that the TCGA-HNSCC cohort has substantial locoregional heterogeneity of tumor sites whereas this was homogenous in our study, which focused only on the oropharyngeal subsite of HNSCC. Strong correlation (Spearman’s ρ >= 0.8; *p* value <0.05) between methylation levels of promoter DMPs of OPSCC tissues included in this study and that of OPSCC specific samples from TCGA-HNSCC study elucidates robustness of our inferences. *HSPB8*, *ID4*, *ZNF471*, *ZNF582*, *ZNF682* are some of the prominent genes which were found to be epigenetically downregulated in both of the studies. Heat shock protein – HSPB8 is known to have aberrant kinase activity and is found silenced through altered DNA methylation in aggressive and drug-resistant melanoma. Restored expression of this gene inhibits tumor growth through the activation of TAK1-dependent death pathways. *HSPB8* is also found to be epigenetically silenced in human prostate cancer, Ewing’s sarcoma cells and in hematological malignancies (Smith et al., 2012). Inhibitor of DNA Binding 4 (ID4) functions primarily as a tumor suppressor gene. *ID4* has been found to be epigenetically dysregulated in various cancer types: leukemia, AML, CLL, ALL, glial neoplasia, gastric cancer, pancreatic cancer, colorectal, lymphoma, cholangiocarcinoma, esophageal, lung and prostate cancers (Patel et al., 2015). Promoter of *ID4* exhibits significantly increased methylation accompanied by loss of gene expression in squamous cell carcinoma compared to normal skin (Ruchusatsawat et al., 2011). Zinc finger proteins ZNF471, ZNF582, ZNF682 are Krüppel-type family of transcription factors mapped to chr19. *ZNF471* acts as a tumor suppressor in gastric cancer by transcriptionally inhibiting downstream targets TFAP2A and PLS3 (Cao et al., 2018). Additionally, this gene has been found to be epigenetically downregulated and associated with poor survival in squamous cell carcinoma of the tongue (Bhat et al., 2017). Thus, our results, along with the OPSCC data of TCGA cohort, provides distinct DNA methylation and gene expression landscapes which might be specific for oropharyngeal cancer.

Further, the discovery of a number of genes that have not been reported in other cancer types suggest the presence of an epigenetic eco system unique to this cancer type. This provides potential targets for development of novel therapeutic interventions and prediction of malignancy. These novel genes, *inter alia*, include *GRAP2*, *NLRP3*, *LST1*, *PIK3R6*, *SPN*, *TSPAN32* which are involved in vital processes such as regulation of cell activation and proliferation (Law et al., 1999; Roliński et al., 1999; Rollinger-Holzinger et al., 2000; Tarrant et al., 2002; Voigt et al., 2006; Yu et al., 2020). These processes are known to be involved in oncogenesis. The validation of these results and further targeted experiments on various stages of cancer development can therefore identify genes which can be used as biomarkers for early diagnosis leading to better treatment outcomes.

The results of our study are important for various reasons. We have focused on oropharyngeal cancer, a subtype of head and neck cancer on which less information exists compared to other subtypes viz. oral and tongue. In particular, we have worked in Meghalaya, located in the North East India, a relatively remote region. This region has higher prevalence of OPSCC compared to other regions in the subcontinent, which is associated with typical risk exposures like chewing of areca nut with betel leaf and tobacco. Interestingly, while 64% of OPSCC tumors have been reported to be HPV positive in TCGA (Viswanathan et al., 2003), we could not detect HPV DNA or RNA in any of the tumors included in our study. This might indicate that in this region of the globe, the overwhelming magnitude of exposure caused by the chewing habits mentioned earlier might render the effect of HPV infection superfluous in causation of OPSCC. We have specifically included paired tumor and adjacent tissue samples from the same patients, which makes our DNA methylation and gene expression results more robust than those that are based on unpaired tumor and normal samples.

Our study suffers from multiple limitations. In particular, we would like to point out that we were only able to include a limited number of patients from whom both tumor and adjacent normal tissue samples in sufficient quantities could be obtained for both DNA methylation array and RNA-Seq analyses. This was primarily caused due to the restricted site of oropharynx, the size of tumors and difficulty in accessing the site. For the same reason, we could not carry out independent candidate gene validation experiments. However, the strengths of our findings are due to the fact that they are based on correlated DNA methylation and gene expression results. We suggest that our results should be validated in an independent cohort of patients located in this region.

## Conclusion

This study is the first integrative attempt to address whole genome methylation and transcriptome analyses of oropharyngeal cancer in the North eastern population of India where the disease is very prevalent. The findings of many “novel” genes that are epigenetically regulated in the development of oropharyngeal cancer can open up new avenues of biomarker discovery not only in this population of NE India but also in the etiology of the disease in other populations.

## Data Availability Statement

The Illumina Infinium Human Methylation 450K microarray data has been submitted to NCBI Gene Expression Omnibus and has been approved with Accession Number GSE136704. The raw paired end FASTQ files of RNA-Seq data were submitted to European Nucleotide Archive (ENA) under the Study ID PRJEB34402.

## Ethics Statement

The studies involving human participants were reviewed and approved by Institutional Ethics Committees of North Eastern Hill University, Shillong, Meghalaya (IECHSP/2015-16 dated 23 June 2017), North Eastern Indira Gandhi Regional Institute of Health and Medical Sciences, Shillong, Meghalaya (NEIGR/IEC/2015/0039 dated 21 April 2016) and National Institute of Biomedical Genomics, Kalyani (Dated 12th June 2015). The patients/participants provided their written informed consent to participate in this study.

## Author Contributions

SrG and AM conceived the study. NS, SrG, and LM coordinated patient recruitment and sample collection. AM, SaG, and AP coordinated data generation and data collation. LM, SaG, AP, and MI undertook analysis and interpretation of data. LM, SaG, AP, AM, and SrG wrote the manuscript. All authors read and approved the final manuscript.

## Conflict of Interest

The authors declare that the research was conducted in the absence of any commercial or financial relationships that could be construed as a potential conflict of interest.

## Abbreviations

cDNA: complementary DNA
CGI: CpG island
DMP: differentially methylated probe
DMR: differentially methylated region
FPKM: fragments per kilobase of transcript per million mapped reads
GO: gene ontology
HCC: hepatocellular carcinoma
HNSCC: head and neck squamous cell carcinoma
KEGG: Kyoto Encyclopedia of Genes and Genomes
MAF: minor allele frequency
OSCC: oral squamous cell carcinoma
OPSCC: oropharyngeal squamous cell carcinoma
RIN: RNA integrity number
rRNA: ribosomal RNA
SCC: squamous cell carcinomas
SWAN: subset quantile within array normalization
TCGA: The Cancer Genome Atlas
TSS: transcription start site.
