# Supplementary material for: Whole Genome DNA Methylation and Gene Expression Profiling of Oropharyngeal Cancer Patients in North-Eastern India: Identification of Epigenetically Altered Gene Expression Reveals Potential Biomarkers
Source: Front Genet. 2020 Oct 8;11:986. doi: 10.3389/fgene.2020.00986 (PMC7578381; doi:10.3389/fgene.2020.00986)
Supplement: Supplementary file 1 [file Data_Sheet_1.docx]

**Supplementary Information**

**In-silico validation of DNA methylation data by orthogonal approach**

An orthogonal DNA methylation analysis pipeline was used to validate differential promoter methylation profile in oropharyngeal carcinoma tissues compared to adjacent normal tissues. Paucity of samples restricted us from further experimental confirmation of our inferences.

Illumina GenomeStudio methylation module was used to process raw intensity data files (*.IDAT) and to obtain beta values. Further downstream analysis was performed using Illumina Methylation Analyzer (IMA) (Wang et al., 2012), an R package. Samples with 75% or more sites having detection p-value > 1e-05 were dropped. Similarly, probes/ sites with a detection p-value > 0.05 present in at least 50% of the samples were removed. Probes mapped to chromosomes X and Y were also excluded. Loci having reported SNPs present within probe were filtered out. Intra array bias was corrected using peak based correction by re-scaling Infinium II data on the basis of the Infinium I data. Inter sample or array variation due to technical artefact was corrected using Quantile normalization. Wilcoxon signed rank test was performed for every loci, taking tumour and adjacent normal data in two different group. A CpG locus considered as differentially methylated having multiple testing corrected (Benjamini-Hochberg) p-value < 0.05 and average |Δβ| ≥ 0.2. For a pre-defined region, mean beta value of all the loci present in that particular region quantifies its methylation status. For identification of significantly differentially methylated region, Wilcoxon signed rank test was performed and p-value adjustment was done by Benjamini and Hochberg multiple testing correction. Cut off for region level test was set to BH adjusted p-value < 0.05.

**Supplementary Figures**


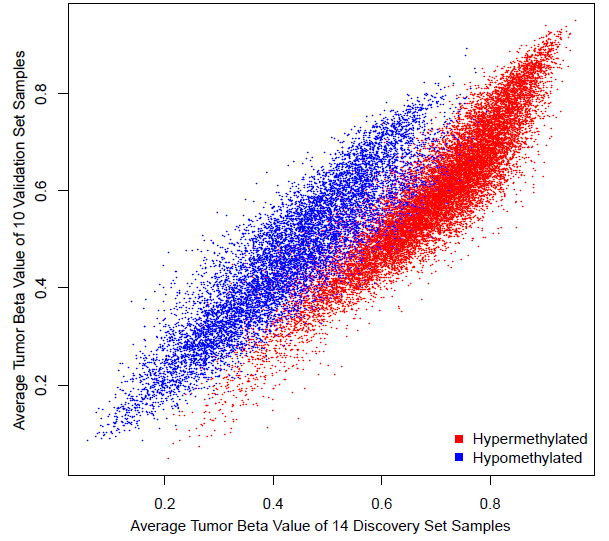


**Supplementary Figure 1: Global methylome profile of OPSCC tissues validated in independent set of tumour tissues of same cancer. Tumour β values of 25,494 DMPs of discovery set (N=14) showed significantly strong correlation (Spearman ρ = 0.86 and P-value < 2.2e-^16^) with that of the β values of same in validation set (N=10).**


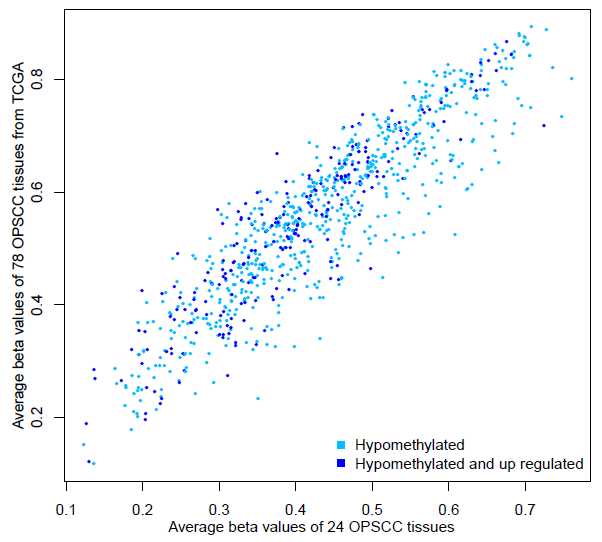

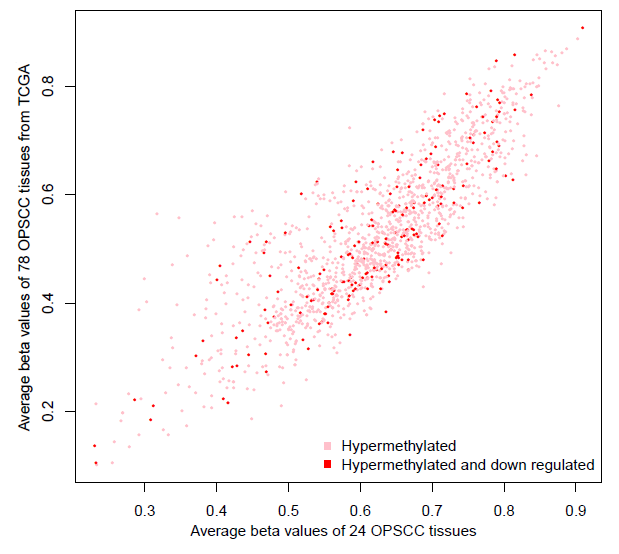


**(B)**

**(A)**

**Supplementary Figure 2: Graphic representation of correlation between 24 OPSCC patients from North Eastern Indian states with publicly available 78 OPSCC patients from TCGA-HNSCC cohort. Tumour β values of 1421 hypermethylated and 895 hypomethylated promoter genes showed strong correlations between two comparable dataset. (A) The hypermethylated genes showed strong correlation (ρ = 0.8; p-value < 2.2x10−16) whereas (B) hypomethylated genes showed even stronger correlation (ρ = 0.9; p-value < 2.2x10−16).**

**References**

1. Wang, D., Yan, L., Hu, Q., et al. (2012) IMA: an R package for high-throughput analysis of Illumina's 450K Infinium methylation data. Bioinformatics. ,28(5):729–730. doi:10.1093/bioinformatics/bts013
